# Waardenburg syndrome

**DOI:** 10.11604/pamj.2023.45.23.38829

**Published:** 2023-05-05

**Authors:** Iyer Lavanya Ramakrishnan, Amar Taksande

**Affiliations:** 1 Department of Pediatrics, Datta Meghe Institute of Higher Education and Research Wardha, Maharashtra, India

**Keywords:** Waardenburg syndrome, forelock, coloboma

## Image in medicine

Waardenburg syndrome (WS) is named after the Dutch ophthalmologist Petrus Johannes Waardenburg, who first described the syndrome in 1947. Waardenburg syndrome type-2 (WS2) is an autosomal disorder. It is a heterogeneous disorder of neural crest cell development with distinct cutaneous manifestations. Waardenburg syndrome is classified into 4 mean phenotypes. This syndrome is a result of multiple gene mutations, of which 6 genes are known. It is an auditory-pigmentary syndrome characteristically showing pigmentary abnormalities of the hair, skin and eyes, often associated with sensorineural hearing loss and absence of dystopia canthorum, which differentiates it from Waardenburg type-1. Mutations in the microphthalmia-associated transcription factor (MITF) gene, which is located on chromosome band 3p14.1-p12.3, cause some cases of WS2 (15%). Our patient here presented at the age of 14 years with ocular abnormalities. On examination, the patient had a white forelock since birth associated with premature graying, right-sided microphthalmia with inferior coloboma of iris on the right eye. The patient had no complaints of diminished hearing. Complete vision and hearing evaluation was done, which was normal. Though not genetically determined, the patient was given a working diagnosis of Waardenburg syndrome type 2. Differential diagnoses that can be considered are Piebaldism, Vogt-Koyanagi-Harada disease or Teitze syndrome.

**Figure 1:**
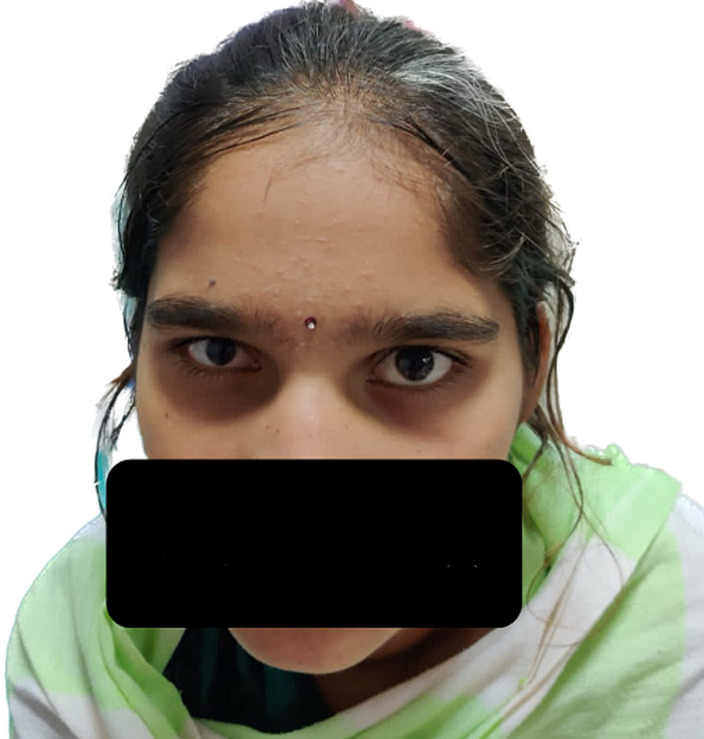
Waardenburg syndrome +

